# Deep learning-Based 3D inpainting of brain MR images

**DOI:** 10.1038/s41598-020-80930-w

**Published:** 2021-01-18

**Authors:** Seung Kwan Kang, Seong A. Shin, Seongho Seo, Min Soo Byun, Dong Young Lee, Yu Kyeong Kim, Dong Soo Lee, Jae Sung Lee

**Affiliations:** 1 Department of Biomedical Sciences, Seoul National University College of Medicine, Seoul, Korea; 2 Department of Electronic Engineering, Pai Chai University, Daejeon, Korea; 3 Institute of Human Behavioral Medicine, Medical Research Center, Seoul National University, Seoul, Korea; 4 Department of Psychiatry, Seoul National University College of Medicine, Seoul, Korea; 5 Department of Nuclear Medicine, SMG-SNU Boramae Medical Center, Seoul, Korea; 6 Department of Nuclear Medicine, Seoul National University College of Medicine, 103 Daehak-ro, Jongno-gu, Seoul, 03080 Republic of Korea

**Keywords:** Alzheimer's disease, Biomedical engineering, Brain, Brain imaging, Magnetic resonance imaging, Three-dimensional imaging, Tomography

## Abstract

The detailed anatomical information of the brain provided by 3D magnetic resonance imaging (MRI) enables various neuroscience research. However, due to the long scan time for 3D MR images, 2D images are mainly obtained in clinical environments. The purpose of this study is to generate 3D images from a sparsely sampled 2D images using an inpainting deep neural network that has a U-net-like structure and DenseNet sub-blocks. To train the network, not only fidelity loss but also perceptual loss based on the VGG network were considered. Various methods were used to assess the overall similarity between the inpainted and original 3D data. In addition, morphological analyzes were performed to investigate whether the inpainted data produced local features similar to the original 3D data. The diagnostic ability using the inpainted data was also evaluated by investigating the pattern of morphological changes in disease groups. Brain anatomy details were efficiently recovered by the proposed neural network. In voxel-based analysis to assess gray matter volume and cortical thickness, differences between the inpainted data and the original 3D data were observed only in small clusters. The proposed method will be useful for utilizing advanced neuroimaging techniques with 2D MRI data.

## Introduction

The detailed anatomical information of the brain provided by T1-weighted magnetic resonance (MR) imaging with high-resolution three-dimensional (3D) sequences enables a variety of brain imaging studies, including quantitative measurements of brain tissue volume and cortical thickness, and classification of images for early diagnosis of disease^1–6^. Moreover, 3D MR images are useful for accurate anatomical localization and quantification of co-registered functional images such as those obtained using positron emission tomography or single-photon emission tomography^7,8^. However, 3D MRI sequences require longer scan times and larger data storage, so 2D images (multiple 2D slices with substantial gaps between them) are commonly acquired for diagnostic purposes in routine clinical practice. This may hinder the use of advanced neuroimage analysis techniques such as voxel-based morphometry and cortical thickness measurement for clinical data evaluation.

Numerous deep learning-based approaches have been proposed in recent years to solve a variety of computer vision problems, with significant results outperforming conventional algorithms^9–14^. This is mainly because the improvement in computational power makes it possible to deal with large data sets based on highly manipulated neural network structures, thereby allowing them to solve complex problems. One of the advantages of deep learning over traditional mathematical and statistical methods is that it is relatively simple to configure the relationship between the input and the output spaces in various situations. Therefore, these methods have been quickly implemented in medical imaging and have achieved remarkable achievements in image classification, segmentation, noise reduction, and image generation^15–26^.

**Figure 1:**
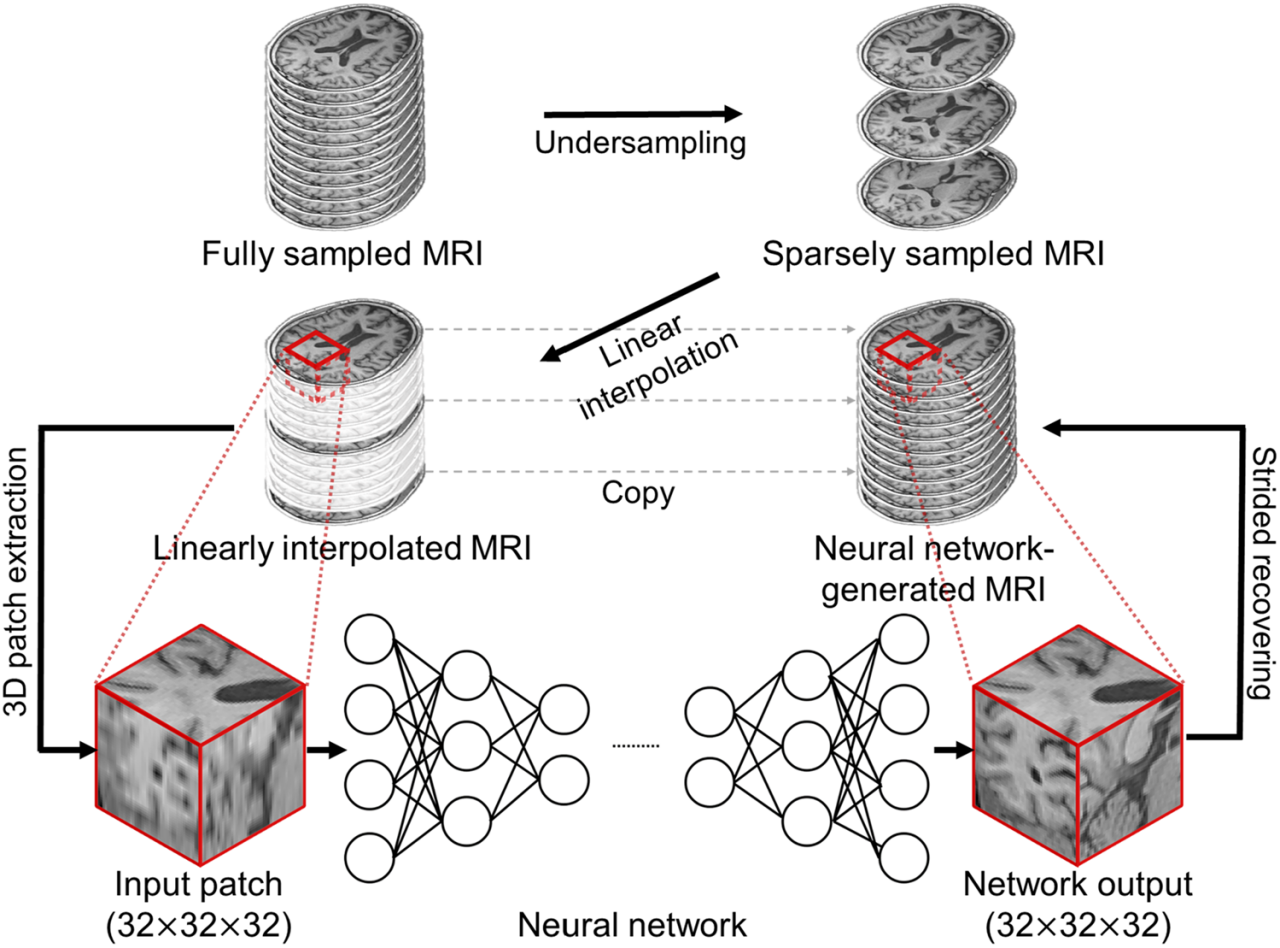
Schematic diagram of the proposed deep learning-based 3D inpainting method for magnetic resonance images.

In this study, we propose a deep learning-based inpainting technique that can generate 3D T1-weighted MR images from sparsely acquired 2D MR images (Fig. 1). The proposed network was trained to produce 3D inpainted MRI from the input that is linearly interpolated from sparsely sampled MR images. This approach allows the generation of 3D images without careful modeling of hand-crafted prior or assumptions about brain anatomy. The similarity between the inpainted and reference images was quantitatively analyzed based on real 3D MR images. We also used a voxel-based morphometry analysis and cortical thickness measurement to assess whether the complex structures of the cerebral cortex were correctly recovered and whether the inpainted data yielded equivalent morphological measurements to the original 3D data (reference image). Furthermore, we analyzed brain atrophy patterns in disease groups to see if we could characterize disease patterns using images generated based on deep learning.

## Methods

### Datasets

We trained and tested deep neural networks using 680 T1-weighted 3D MR images obtained from the Korean Brain Aging Study for Early Diagnosis and Prediction of Alzheimer’s Disease (KBASE)^27^. Institutional Review Board of Seoul National University Hospital approved the study and all study participants provided informed consent. All experiments were conducted in accordance with relevant guidelines and regulations. T1-weighted 3D MR images of the subjects were obtained using a PET/MRI scanner (3-T, Biograph mMR, Siemens, Knoxville, Tennessee, USA). The imaging parameters were as follows: 208 slices with sagittal acquisition; slice thickness: 1 mm; repetition time: 1670 ms; echo time: 1.89 ms; flip angle: 9°; acquisition matrix: 208 × 256 × 256; and voxel size: 1.0 mm × 0.98 mm × 0.98 mm. Of the 680 datasets, 527 were used to train the neural network and the remaining 153 were used to test the trained network. Of the 153 datasets, 97 were normal (NL) elderly, 36 mild cognitive impairment (MCI) patients, and 20 Alzheimer’s disease (AD) patients (Table 1).

**Table 1:** Demographic characteristics of subjects: normal (NL) elderly, mild cognitive impairment (MCI) patients, and Alzheimer’s disease (AD) patients.

Training set	NL	MCI	AD
*N*	338	117	72
Age (years)	62.4 ± 15.3	73.68 ± 6.9	72.56 ± 8.2
Gender (% female)	51.2%	65.0%	69.4%
Test set			
*N*	97	36	20
Age (years)	71.6 ± 8.0	72.7 ± 6.3	73.55 ± 8.6
Gender (% female)	60.8%	61.1%	65.0%

### Neural network

#### Proposed CNN architecture

**Figure 2:**
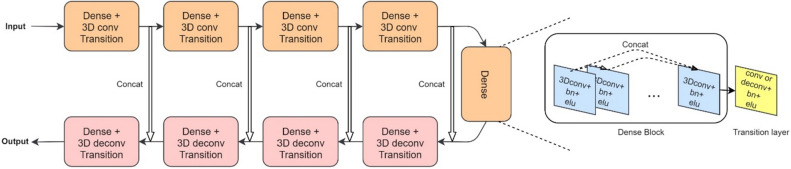
The architecture of the proposed neural network. A detailed explanation of the dense blocks and transition layers is shown on the right. All convolutions (conv) are calculated in a 3D manner, followed by batch renormalization (bn) and exponential linear units (elu) activation functions. The size of the input and output image patches is 32 × 32 × 32. deconv: deconvolution. concat: concatenation.

Figure 2 shows the detailed structure of the proposed network consisting of nine dense blocks^28^ and transition layers (Supplementary Table 1). Each dense block with five convolutional layers is followed by a transition layer, yielding four blocks with a strided convolution transition layer, one without any transition layer, and four with a deconvolution transition layer. The convolutional layers retain the features of the previous layers by concatenating all the passing layers as described in the following equation:

1 $$ {\varvec{x}}_{l} = H_{l} \left( {\left[ {{\varvec{x}}_{0} ,{\varvec{x}}_{1} , \ldots ,{\varvec{x}}_{l - 1} } \right]} \right), $$

where ${{\varvec{x}}}_{i}$ represents the feature map of the $i$ th layer and ${H}_{l}$ represents the composition of the activation function (exponential linear unit) and the batch normalization of the *l*th layer^29^. As shown in Fig. 2 and Eq. (1), each convolution operation in a dense block contains all previous outputs through concatenation and produces the same number of channels, so concatenation from all previous layers increases the number of input channels in the next layer. Therefore, we applied a 1 × 1 × 1 convolution followed by a 3 × 3 × 3 convolution to compress the data. The first dense block layer has 16 channels. The transition layers after the first four dense blocks subsample the images using strided (2 × 2 × 2) convolution to achieve a larger receptive field, whereas in the last four dense blocks, deconvolution (transposed convolution) transition layers follow the convolutional blocks. Features computed from the strided convolutional stages of the dense blocks were concatenated with those from the deconvolution dense blocks to ensure that the same dimension is maintained in the convolutional stage, enabling better backpropagation of the gradients. The architecture described is similar to that of the well-known U-Net^30^.

#### Training details

As shown in Fig. 1, the reference images were sampled every fifth slice in the axial direction and then linearly interpolated. For the training datasets, non-brain tissue was excluded using a brain extraction tool (http://www.nitrc.org/projects/mricron). Input voxel patches of size 32 × 32 × 32 were extracted from the linearly interpolated data for training with a stride of 16 if the center of the patch was in the extracted brain region. We normalized each MR image intensity to a range of − 1 to 1^31,32^. The normalization factor was saved and used to rescale the image after reconstructing 3D image. A total of 439,479 training patches were used for network training with a batch size of 12. After training, the neural network was evaluated using test patches extracted with a stride of 24 and eight overlapping voxels. The overlapping voxels were averaged while reconstructing the inpainted 3D image from the patches. The initial learning rate was set to 0.0003, which decreased to 10% of the initial learning rate after 20 epochs. The network was trained for 60 epochs on a computer with an Intel i7-8770 processor and a Nvidia Geforce GTX 1080ti graphics card.

#### Loss functions

To train the network, not only fidelity loss but also perceptual loss based on VGG network were considered^31^. Accordingly, the total loss was:

2 $$L={L}_{fid}+{\gamma }_{1}{L}_{per}$$

where,

3 $$ L_{fid} = \frac{1}{{mn_{v} }}\mathop \sum \limits_{i = 1}^{m} \left\| {{\varvec{y}}_{ }^{i} - f_{ } \left( {{\varvec{x}}_{s}^{i} } \right)} \right\|, $$

4 $$ L_{per} = \frac{1}{m}\mathop \sum \limits_{i = 1}^{m} \left\| {\phi \left( {{\varvec{y}}^{i} } \right) - \phi \left( {f\left( {{\varvec{x}}_{s}^{i} } \right)} \right)_{2}^{2} } \right\|, $$

$m$ is the batch size, ${\varvec{y}}$ is the vector of reference data, ${{\varvec{x}}}_{s}$ is the sparsely sampled input, and $f$ is the neural network. ${L}_{fid}$ is the fidelity loss reducing the content difference between the reference and the neural network output, ${L}_{per}$ is the perceptual loss in feature space^33^, $\phi$ is the intermediate feature map of a particular neural network, and ${\gamma }_{1}$ is the tuning parameter for the loss functions. In this study, the fourth convolutional layer in the VGG19 network was used as $\phi$ feature map^12^. Given that the VGG19 network requires a single RGB image composed of three channels as input, the 16th sagittal, coronal, and transaxial slices of the 3D input patch were supplied as inputs to the pre-trained VGG19 network. The perceptual loss was evaluated using only the central slices of the input patch for fast network training. The input to the VGG19 was upscaled from 32 × 32 pixels to 224 × 224 pixels using a linear interpolation method so that the size of the extracted slices matched that of VGG19. The tuning parameter ${\gamma }_{1}$ was set to 10^−6^.

### Comparison

For comparison, we also implemented the well-known U-Net using the same numbers of convolutional filters and pooling layers as described in the original paper^30^, but using a different convolution operation dimension (3D instead of 2D) of and input and output sizes (both 32 × 32 × 32). The U-Net was trained and tested using the same dataset used for the proposed network.

### Similarity evaluations

For the test dataset, three quantitative measures were used to evaluate the similarity between the reference image (original 3D image) and the inpainted images (linear interpolated data and neural network-generated data). The similarity assessment was applied to the brain regions defined by the brain mask produced using the brain extraction tool^34^. Peak signal-to-noise ratio (PSNR), structural similarity (SSIM), and high-frequency error norm (HFEN) were calculated using the following equations^35,36^.

5 $$ PSNR = 10\log_{10} \left( {\frac{{max\left( {I_{y} } \right)}}{MSE}} \right), $$

6 $$SSIM=\frac{\left(2{\mu }_{x}{\mu }_{y}+{c}_{1}\right)\left(2{\sigma }_{xy}+{c}_{2}\right)}{({\mu }_{x}^{2}+{\mu }_{y}^{2}+{c}_{1})({\sigma }_{x}^{2}+{\sigma }_{y}^{2}+{c}_{2})},$$

7 $$HFEN=\frac{{\Vert LoG\left({I}_{x}\right)-LoG\left({I}_{y}\right)\Vert }_{2}}{{\Vert LoG\left({I}_{y}\right)\Vert }_{2}},$$

where ${I}_{x}$ is the test image, ${I}_{y}$ is the reference image, and $MSE$ is the mean squared error between them. Here, ${\mu }_{(\bullet )}$ and ${\sigma }_{(\bullet )}$ denote the mean and variance or covariance of two images, and ${c}_{1}={\left(0.01\times d\right)}^{2}$ and ${c}_{2}={\left(0.03\times d\right)}^{2}$. $LoG(\bullet )$ denotes the Laplacian of 3D Gaussian filter function with the kernel size of 15 × 15 × 15.

### Voxel-based morphometry analysis and cortical thickness measurements

Different brain tissue types (gray matter (GM), white matter (WM), and cerebrospinal fluid (CSF)) were segmented from the reference and inpainted images using Statistical Parametric Mapping 12 (SPM12; http://www.fil.ion.ac.uk/spm)^37^. The segmented images were then quantitatively examined using Dice similarity coefficient^38^ to compare the similarity between the reference and inpainted data.

To assess the accuracy of the volumetric measures estimated from the inpainted data, a voxel-based morphometry method based on the SPM12 pipeline was applied to the GM images. Inter-subject registration of GM images was performed based on nonlinear deformation using a fast diffeomorphic image registration toolbox^39^, and the images were modulated to preserve the tissue volume. The images were then smoothed with a Gaussian kernel with a full width at half maximum of 10 mm. The GM volume images of the inpainted data were then compared to the reference data in a voxel-wise manner using *t*-test with the total intracranial volume as a covariate. The statistical threshold was set to *p*-value of 0.005 (corrected for family-wise error) and cluster extent of 100 voxels. The GM volumes in preselected regions (frontal, lateral parietal, posterior cingulate-precuneus, occipital, lateral temporal, hippocampus, caudate, and putamen regions) were also estimated using an automatic anatomic labelling algorithm^40,41^ to evaluate the correlation between the inpainted and reference data.

Cortical thickness measurements were performed using the automated image preprocessing pipeline of Freesurfer (v6.0.0; http://surfer.nmr.mgh.harvard.edu/). The processing pipeline included skull stripping, nonlinear registration, subcortical segmentation, cortical surface reconstruction, and parcellation^42–45^. The output of the automated processes was visually inspected for obvious errors. Instead of manually correcting for the errors, 15 subjects were excluded from the statistical analysis. Cortical thickness values were smoothened via surface-based smoothing with a Gaussian kernel with a full width at half maximum of 5 mm. Moreover, a two-sample *t-*test was performed to detect brain regions with differences in cortical thickness between the inpainted and reference data. Results were considered significant if survived at false-discovery rate corrected *p*-value of 0.05 at cluster level. The correlation of cortical thickness between the inpainted and reference data was also analyzed.

Finally, we performed voxel-wise comparisons of the GM volume and cortical thickness between NL and MCI/AD groups to investigate whether the inpainted images yield similar patterns of morphological abnormalities similar to those shown by the reference data in the MCI and AD groups.

## Results

### Similarity between inpainted and reference images

**Figure 3:**
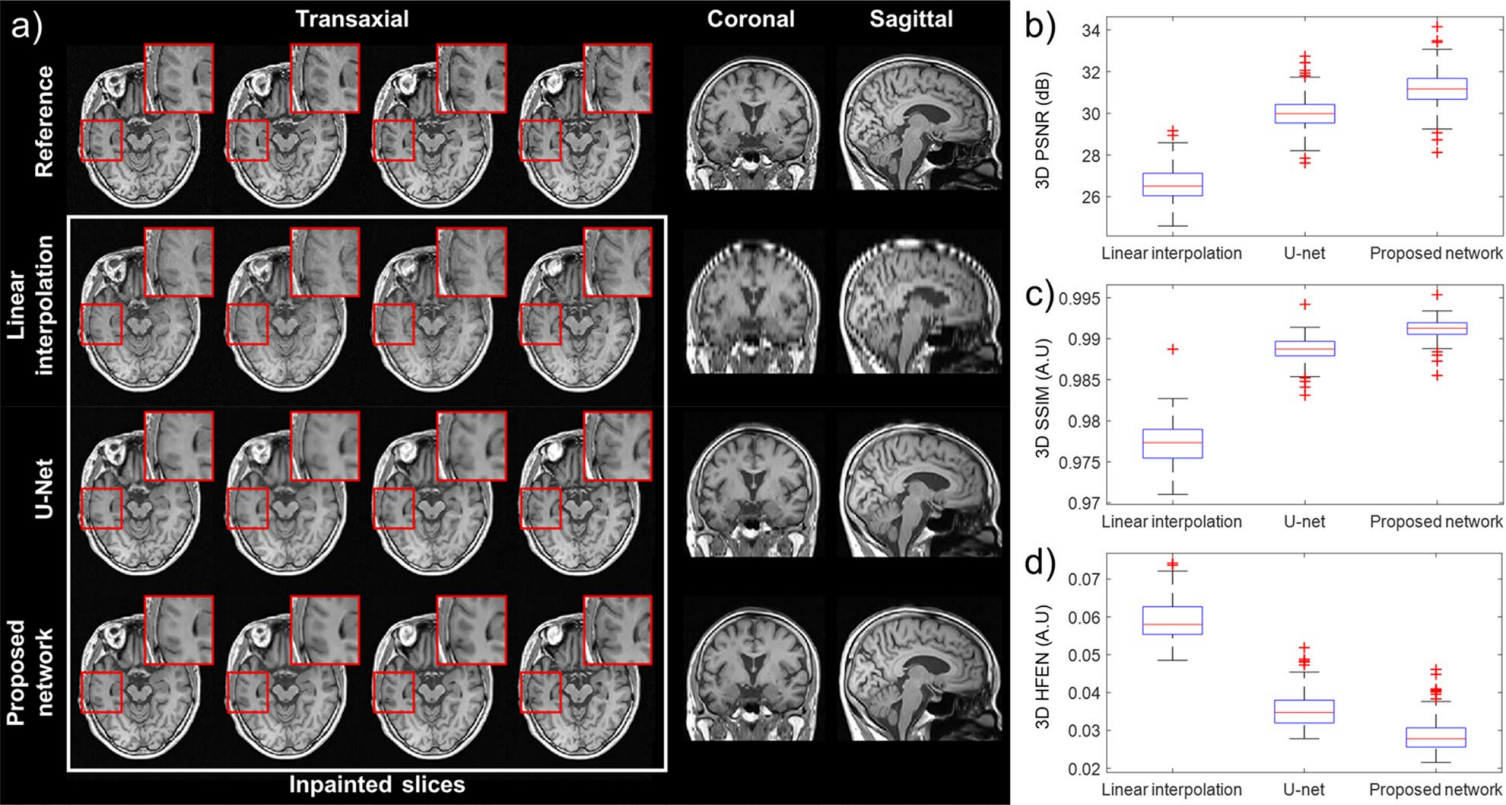
Comparison of inpainted images to the original 3D MR images (reference). (**a**) Each column shows the 137th to 140th transaxial slices, the 95th coronal slice, and the 125th sagittal slice. Results of PSNR (**b**), SSIM (**c**) and HFEN (**d**) evaluation of the accuracy of the different inpainting methods (linear interpolation, U-Net and proposed network) relative to reference data are shown on the left.

The left columns of Fig. 3a show the transaxial slices of the neural network-generated data (third and fourth rows) and the linearly interpolated data^46^ (second row) compared to the reference data (top row). The proposed neural network has well recovered the anatomical details in the brain cortices and ventricles and showed high contrast between GM and WM. In particular, the output of the proposed method had less blurred structures and edges than that of U-Net. On the other hand, the linear interpolation method led to interpolation artifacts and image blurring. The artifacts and blurring most pronounced around the ventricles have resulted in high distortion in the coronal and sagittal planes.

PSNR, SSIM, and HFEN between the inpainted and reference data were calculated in a 3D manner for 153 test datasets. As shown in Fig. 3b–d, all metrics indicate that the images generated using the proposed neural network method are more similar to the reference than the images generated using the conventional linear interpolation method and U-Net.

**Figure 4:**
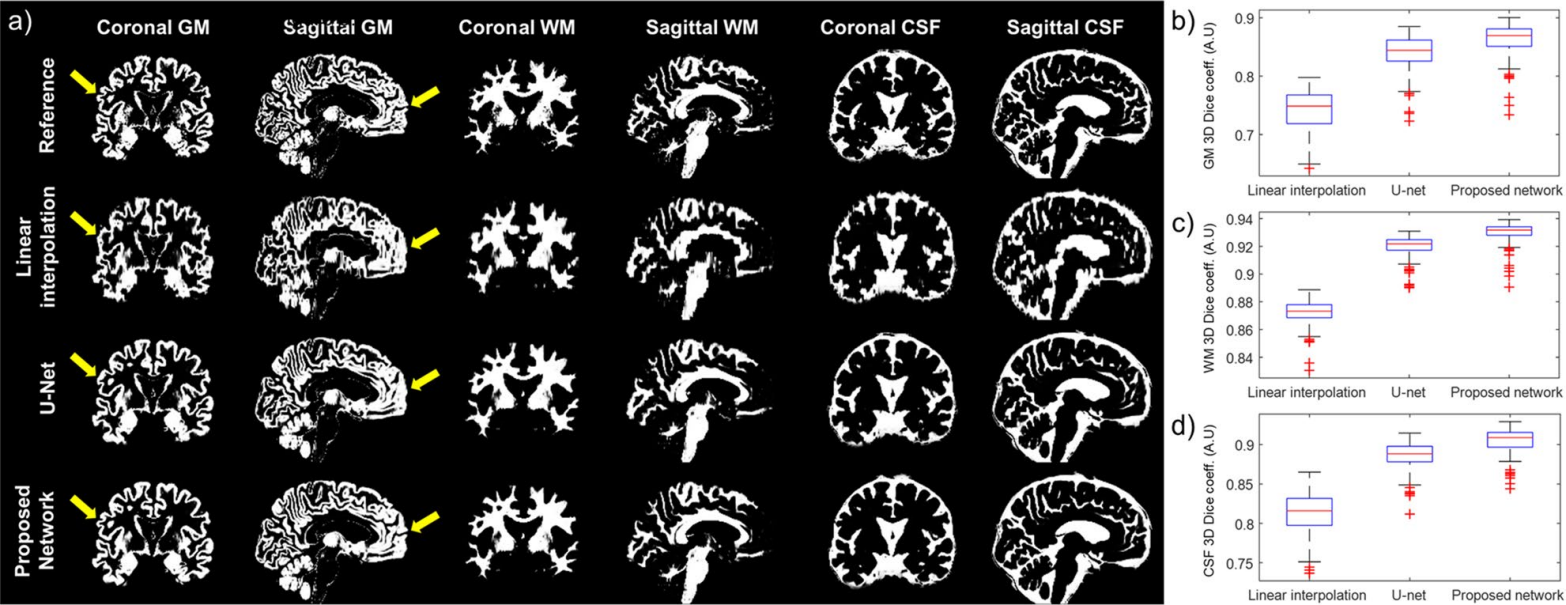
Gray matter (GM), white matter (WM), and cerebrospinal fluid (CSF) segments of a representative image. (**a**) The areas indicated by arrows show poor recovery of the GM segments in linearly interpolated images (second row) and U-Net images (third row) compared to the proposed neural network-generated images (fourth row). 3D Dice coefficients between reference (original 3D MRI) and inpainted data in GM (**b**), WM (**c**), and CSF (**d**) are shown on the left.

The superiority of the proposed method was also evident in the segmentation of GM in the inpainted images. Figure 4a shows the coronal and sagittal planes of segmented GM, WM, and CSF images. In the GM maps, the cortical gyri were not fully recovered when using linear interpolation and U-Net as indicated by the arrows in the figure. However, the GM maps generated by the proposed neural network method are almost identical to the reference images. Figure 4b–d show the Dice similarity coefficients relating to the inpainted and reference images for the different tissue maps, indicating that the proposed method outperformed the conventional interpolation method and U-Net.

### Voxel-based morphometry and cortical thickness analyses

**Figure 5:**
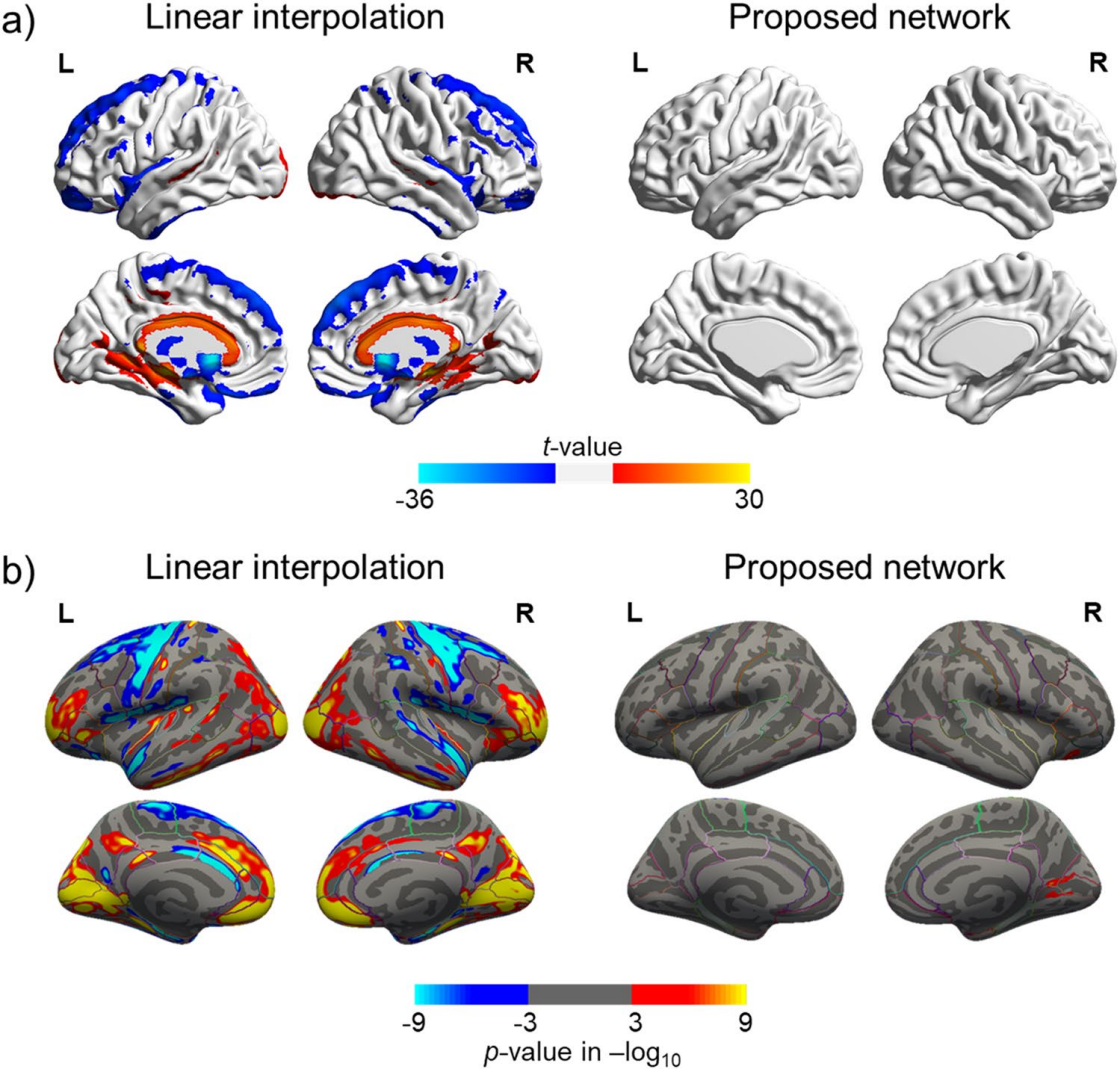
Voxel-wise comparison of inpainted data to the reference data (original 3D MRI). (**a**) Gray matter volume difference. (**b**) Cortical thickness difference. Red indicates overestimation, and blue indicates underestimation relative to the reference data.

Compared to the reference data, the proposed neural network-generated inpainted data showed no significant differences in the GM volume estimates (Fig. 5a and supplementary Fig. 1). On the other hand, the linearly interpolated data showed overestimation in the medial areas of the brain and underestimation in the bilateral frontal and occipital lobe, insula, striatum, and thalamus with respect to the GM volume. Similarly, as shown in Fig. 5b, when using the neural network-generated data, differences in the cortical thickness were found only in the small clusters in the medial occipital lobe. On the other hand, the cortical thickness was overestimated in the cingulate, bilateral occipital lobe, prefrontal cortex, and temporal lobe and underestimated in the bilateral frontal lobe and lateral temporal lobe when using the linearly interpolated data.

**Figure 6:**
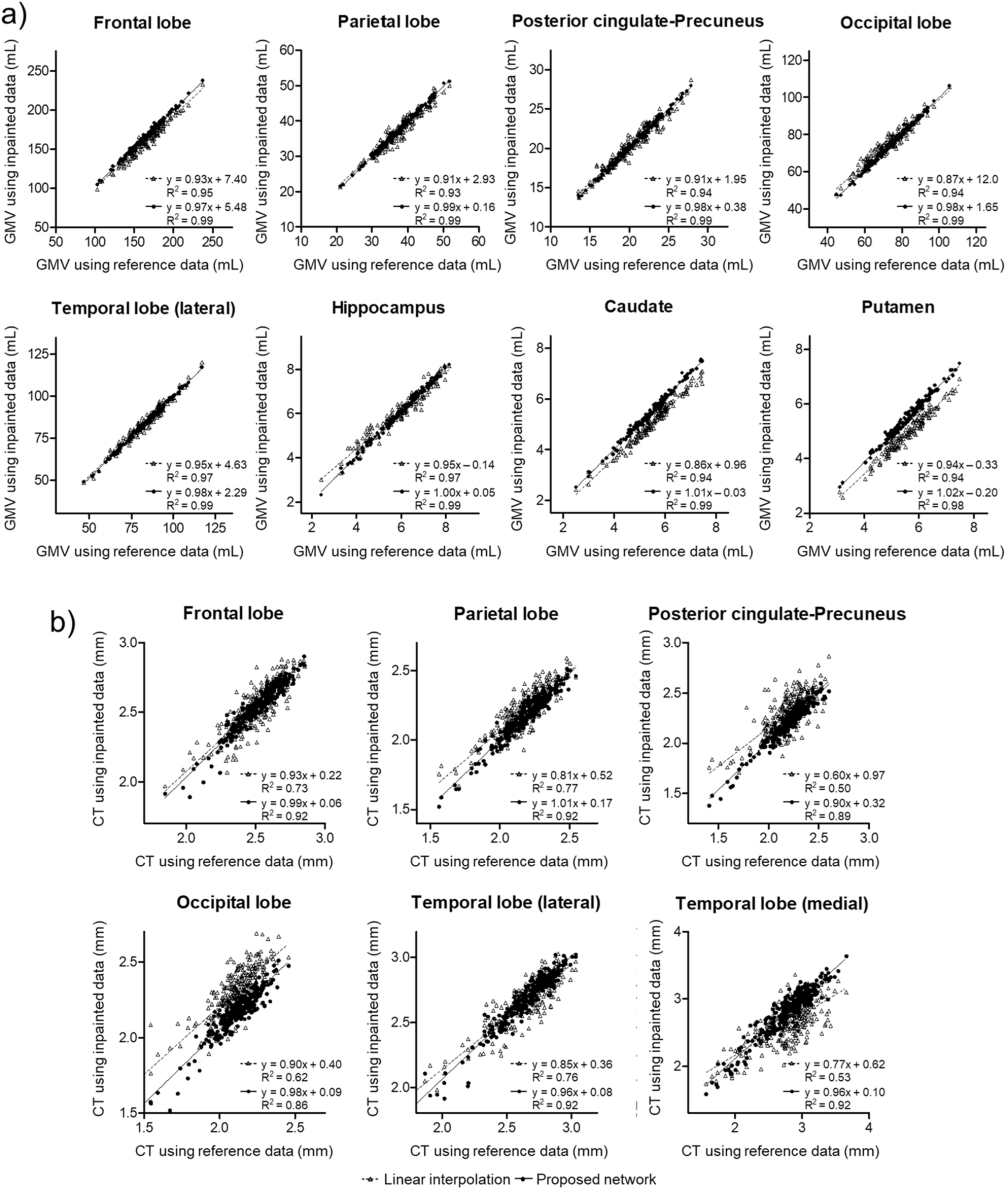
Correlations of regional gray matter volume (**a**) or cortical thickness (**b**) between reference (*x*-axis) and inpainted (*y*-axis) data. Solid lines with a filled circle are for the proposed neural network, and dashed lines with an empty triangle are for linear interpolation.

The individual regional GM volumes estimated using the neural network-generated data are highly correlated with the volumes estimated using the reference data (Fig. 6a). For all the regions analyzed in the study, the volumetric measures obtained using the neural network-generated data showed a higher correlation than the measures obtained with the linearly interpolated data. Regional cortical thicknesses obtained with the inpainted and reference data were also compared (Fig. 6b). The correlation between the neural network-generated and reference data was higher in all regions.

**Figure 7:**
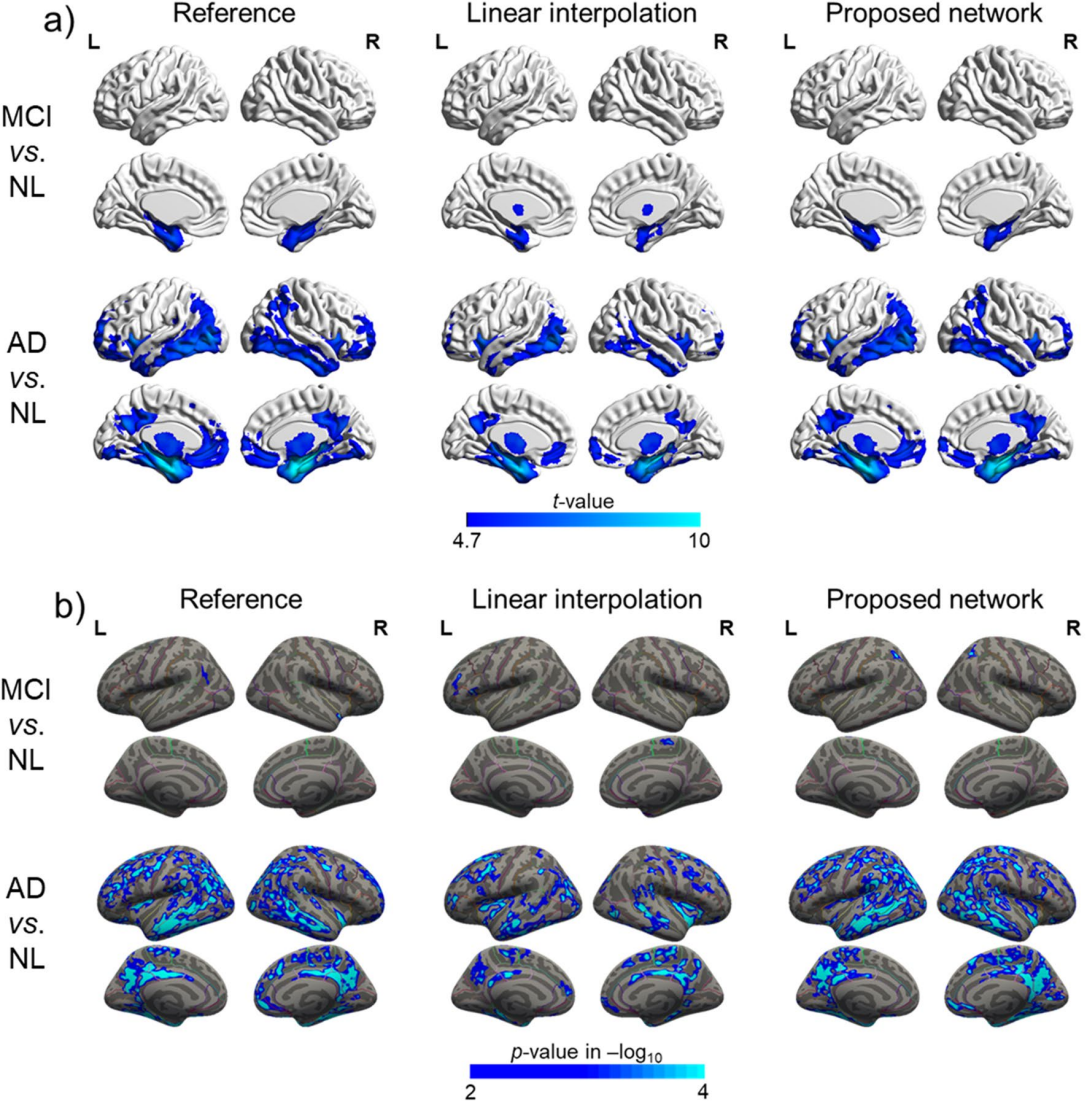
Group analysis among NL, MCI and AD. (**a**) Gray matter volume. (**b**) Cortical thickness.

Figure 7a and Supplementary Fig. 2 shows the patterns of the pathological gray matter volume reductions in the MCI and AD groups compared to the NL group identified using the inpainted and reference data. The reference data and inpainted data based on the proposed network showed GM volume reductions in the bilateral medial temporal lobe in the MCI group, whereas the bilateral frontal, parietal, and temporal lobes were significantly affected in the AD group. As shown in Fig. 7b, the atrophy patterns observed in the MCI and AD groups using the neural network-generated data were more similar to the patterns found using the reference data. In both the inpainted data using the proposed method and reference data, the cortical thickness of the AD group was significantly decreased in the temporal, parietal, dorsolateral frontal, and posterior cingulate cortices. However, the thinning of the cortex in the MCI group was almost undetectable.

## Discussion

This study proposes a deep neural network approach for the 3D inpainting of sparsely sampled human brain MR images. We observed that the brain structure was well recovered using the proposed neural network method without significant interpolation artifacts or noise. Several similarity measures showed that the inpainted images obtained using the proposed method were more similar to the original reference images than those obtained using the linear interpolation method and U-Net. Furthermore, voxel-wise comparison analyses of the GM volume and cortical thickness demonstrated that the 3D MR images generated using the proposed neural network outperformed the conventional linearly interpolated images in morphometric measure estimation. Additionally, the cortical atrophy patterns in patient groups identified using neural network-generated data resembled the patterns found using the reference data, reflecting the potential implications of the proposed method for clinical and neuroimaging research.

In comparison with previously suggested medical image interpolation and super resolution technologies^47–51^, the proposed method has the advantage that no prior knowledge regarding the body or organ shapes, or other additional information is required. The methods based on partial differential equations, such as the one proposed by Li et al. assume that the objects reconstructed from the sparsely sampled slices are smooth^51^. In addition, these methods typically require an iterative updating process and careful tuning of the regularization parameters. Integrating information obtained from multiple pulse sequences is another widely used method for conducting inpainting or performing super-resolution in MR images. The subpixel shift with multiple acquisitions and iterative back projection is an example of this approach^47^. Another proposed method employs non-local means between prior high-resolution images and nearest-neighbor interpolated low-resolution images acquired using multiple different pulse sequences^48^. A recently proposed method employs a sparse representation framework using multiple orthogonal anisotropic resolution scans^49,50^. On the contrary, the proposed deep learning approach yields 3D MR images only from the sparsely sampled 2D images given as an input to the trained neural network. Although the proposed network conducted successful inpainting and yielded better performance than U-Net, the current setup might be sub-optimal because numerous network models are surpassing the U-Net in many other applications^28,52–54^. Comprehensive comparisons with state-of-the-art networks are needed as future work.

The voxel-based analyses of the GM volume and cortical thickness offers the reliability of employing inpainted images for neuroimaging research. Moreover, using the network-generated images, the individual regional GM volume estimates including the subcortical structures highly correlated with the GM volume estimates obtained using the reference data. Subcortical structures, such as the hippocampus and striatum, generally show high inter-individual anatomical variability and are highly affected in diseased conditions, which makes them prone to produce reconstruction errors, diminishing accuracy in 3D interpolation processes. Our results demonstrated that the recovery of the 3D brain images using the proposed method is reliable for clinical and neuroimaging research applications. The acquisition of 3D MR images using the proposed method offers several advantages when only 2D slices are available. The utilization of 3D MR images facilitates the diagnosis of neurodegenerative diseases since regional volume measurements often serve as an important neuroimaging biomarker in many neurodegenerative diseases (for example, hippocampal volume in dementia and striatal volume in movement disorders^55–58^). Moreover, 3D images can aid in monitoring disease progression and predicting prognosis. The pattern of progressive cerebral volume reduction, which precedes the onset of a disease, can be detected using the inpainted 3D images^56,59^. For example, cerebral atrophy pattern lies in line with the pathological staging scheme in Alzheimer’s disease, and allows the early detection of dementia and prediction of the disease progression. The inpainting of brain images can also be used for 3D MRI-guided neurosurgical planning and intraoperative navigation as they enable 3D visualization of a surgical area, whereas 2D slices only offer a limited view. Using 3D data, morphometric measurements, such as cortical thickness and tissue volume, can be performed and assessed as shown in this study. Furthermore, the 3D inpainting technique would enable the application of recently proposed automated methods used to classify brain images to ascertain the type of disease as well as for the prediction of diseases based on machine learning.

Although the proposed 3D SR method could recover brain structures in unscanned slices well, further investigation regarding its ability to detect small lesions should be conducted. However, it is unlikely that the proposed method can recover lesions that are not included in the scanned 2D slices. Supplementary Fig. 3 shows an example of how we can utilize the proposed method in the clinical setting. In the typical simultaneous head-and-neck PET/MRI studies, only the sparsely sampled transaxial 2D MRI slices are acquired although PET images are reconstructed with almost isotropic voxel size. The 3D MR images with the same voxel size as PET generated using the proposed method will be useful for the anatomical localization of PET findings in MR images and other various tasks, such as tumor volume estimation and MRI-guided PET reconstruction.

As a future study, further investigation on the generalization power of the proposed method across sites and countries will be necessary. Supplementary Fig. 4 shows the inpainting results of T2* and FLAIR MR images available in the Alzheimer’s Disease Neuroimaging Initiative (ADNI) dataset. They were obtained by applying the proposed deep neural network trained using only the T1 MR images obtained from our institute, highlighting the highly potential generalization power of our method.

In this study, we fixed the sampling interval and offset to five slices and one slice, respectively. The effect of offset (the first slice number for slice sampling) doesn’t seem to be considerable as shown in Supplementary Fig. 5, which depicts the PSNR values obtained from tests conducted with the networks trained with a single offset (= 1) and multiple other offsets (= 1, 2, …, 5). Further studies based on transfer learning for different numbers of interleaving slices will make this method more useful. In this study, we only considered filling the gaps of transaxial slices, but applications in other directions are also possible. A simple way to deal with various missing data conditions is to train the network with a mixed data set containing different intervals in different directions.

A limitation of the proposed method is the boundary artifacts caused by patch-based learning. Our task was to fill the missing transaxial slices, and unwanted boundaries were observed in the coronal and sagittal planes. The proposed network showed fewer artifacts than the U-net (Supplementary Fig. 6). A potential reason is that the given slices copied from the reference image did not produce a difference. Moreover, the difference between training and test set can make the problem worse. To reduce this artifact, we can use more patches with a smaller number of strides. Alternatively, we can have a larger number of voxels overlap to make the boundary smoother. However, the former option can increase training inefficiencies, resulting in extended learning time, and the latter requires more time to reconstruct the 3D images from 2D slices. There is a recent report that the generative model and attention gates applied to the inpainting of natural images reduced boundary artifacts compared to the deterministic model^53^. This strategy could also be used in medical imaging to reduce these boundary artifacts.

The number of the training set was selected heuristically (527 out of 680 images). This is another limitation of this study that can be improved with rigorous dataset determination. For example, we can examine the age and diagnosis of the entire dataset and divide it into training and test set to have similar distributions. We only used three central slices of the input patch to calculate the perceptual loss because the VGG network used for assessing the perceptual loss receives only three-channel 2D images as input. Although the incorporation of perceptual loss sharped the edges of the network output, the effect was not dominant. We need to investigate whether the perceptual loss based on a network trained in 3D will improve network performance.

## Conclusions

In this study, we proposed a deep neural network for generating inpainted 3D MR images from intermittently sampled 2D images. Patch-based training of the network was performed by simultaneously minimizing the fidelity and perceptual losses for a large dataset. Through quantitative and qualitative evaluation, it was shown that the proposed method outperformed the conventional linear interpolation method in terms of the similarity between the inpainted and reference images and the validity of the inpainted images in the morphometric measurements.

## Supplementary Information

Supplementary Legends.

Supplementary Figure 1.

Supplementary Figure 2.

Supplementary Figure 3.

Supplementary Figure 4.

Supplementary Figure 5.

Supplementary Figure 6.

Supplementary Table 1.

## Data Availability

The data that support the findings of this study are available on request from the corresponding author. The data are not publicly available due to privacy or ethical restrictions.
