# Supplementary material for: Deep learning-Based 3D inpainting of brain MR images
Source: Sci Rep. 2021 Jan 18;11:1673. doi: 10.1038/s41598-020-80930-w (PMC7814079; doi:10.1038/s41598-020-80930-w)
Supplement: Supplementary file 1 — Supplementary Legends. [file 41598_2020_80930_MOESM1_ESM.pdf]

SUPPLEMENTARY FIGURE LEGENDS

SUPPLEMENTARY FIGURE 1. Gray matter volume difference between U-net and the reference.

SUPPLEMENTARY FIGURE 2. Group analysis for gray matter volume among NL, MCI and AD using the U-net output. (a) MCI vs. NL. (b) AD vs. NL.

SUPPLEMENTARY FIGURE 3. 3D MRI inpainting for PET finding localization in simultaneous PET/MRI study.

SUPPLEMENTARY FIGURE 4. Application of proposed inpainting method to ADNI. Linear interpolation (left) and proposed method (right).

SUPPLEMENTARY FIGURE 5. Comparison of the PSNR obtained using the proposed networks trained with a single offset and multiple offsets (1–5).

SUPPLEMENTARY FIGURE 6. Difference map between the reference and inpainted image.
