# Supplementary figures and images for: Deep learning-Based 3D inpainting of brain MR images
Source: Sci Rep. 2021 Jan 18;11:1673. doi: 10.1038/s41598-020-80930-w (PMC7814079; doi:10.1038/s41598-020-80930-w)

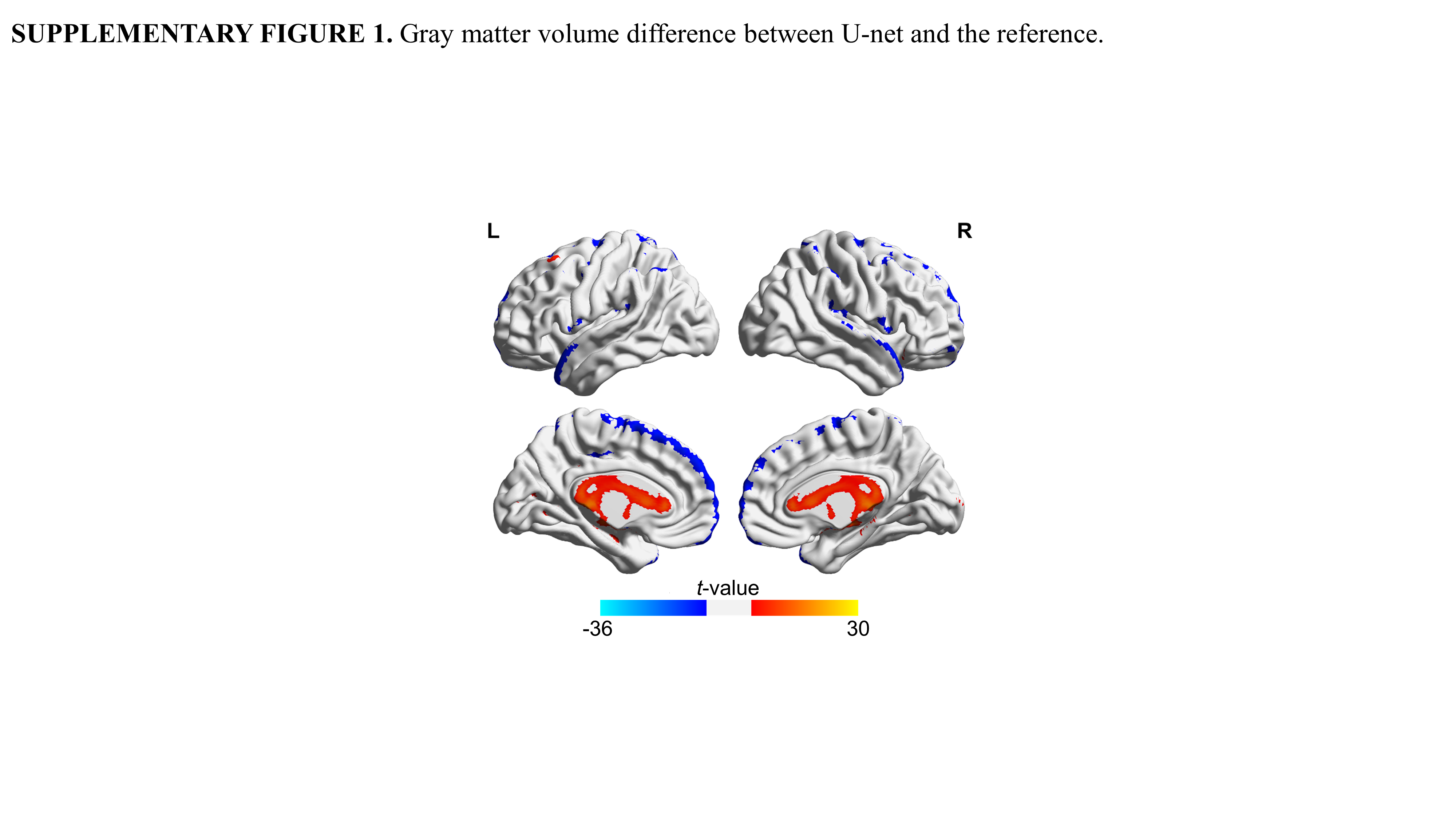

Supplement: Supplementary file 2 — Supplementary Figure 1. [file 41598_2020_80930_MOESM2_ESM.tif]

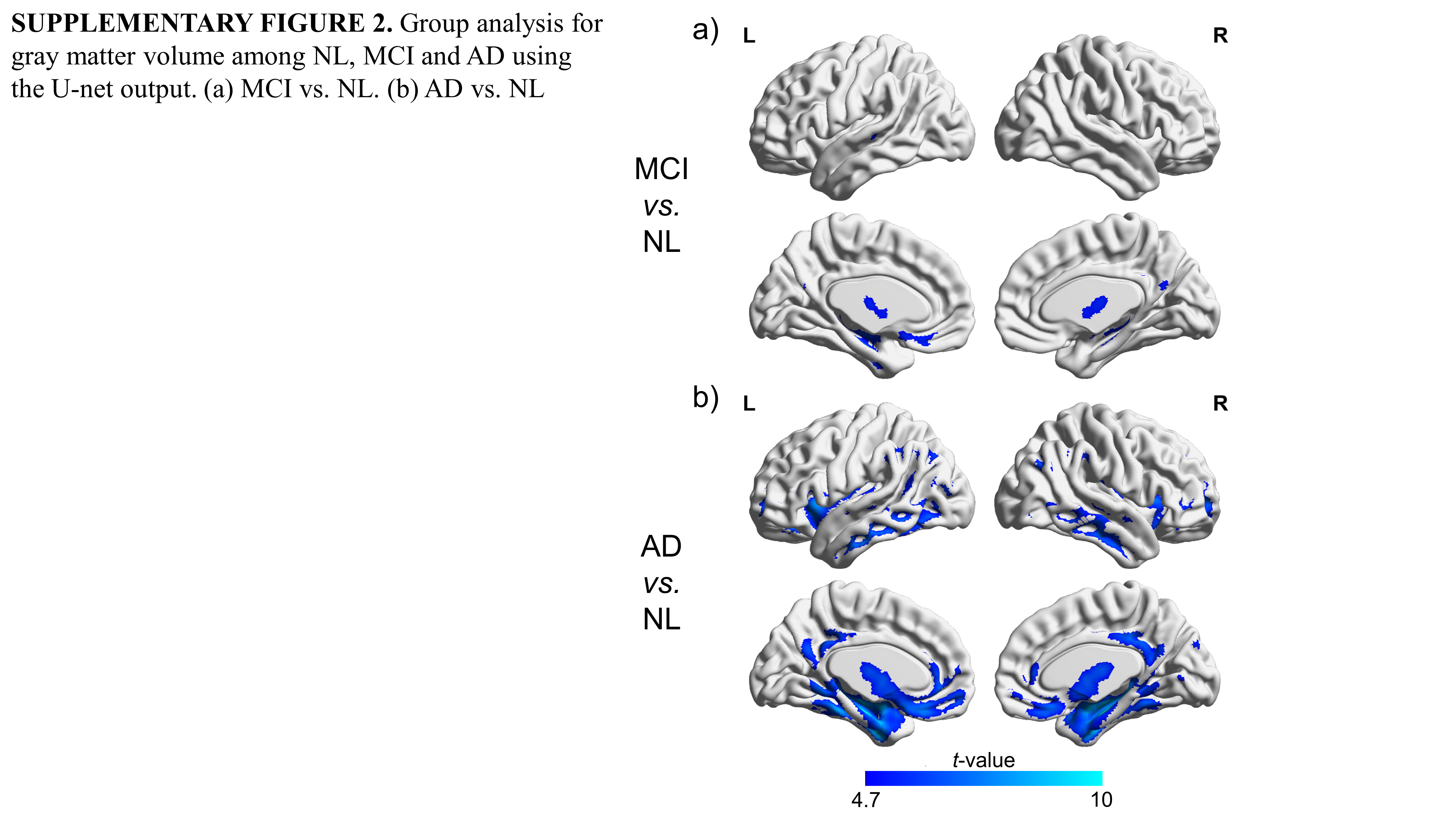

Supplement: Supplementary file 3 — Supplementary Figure 2. [file 41598_2020_80930_MOESM3_ESM.tif]

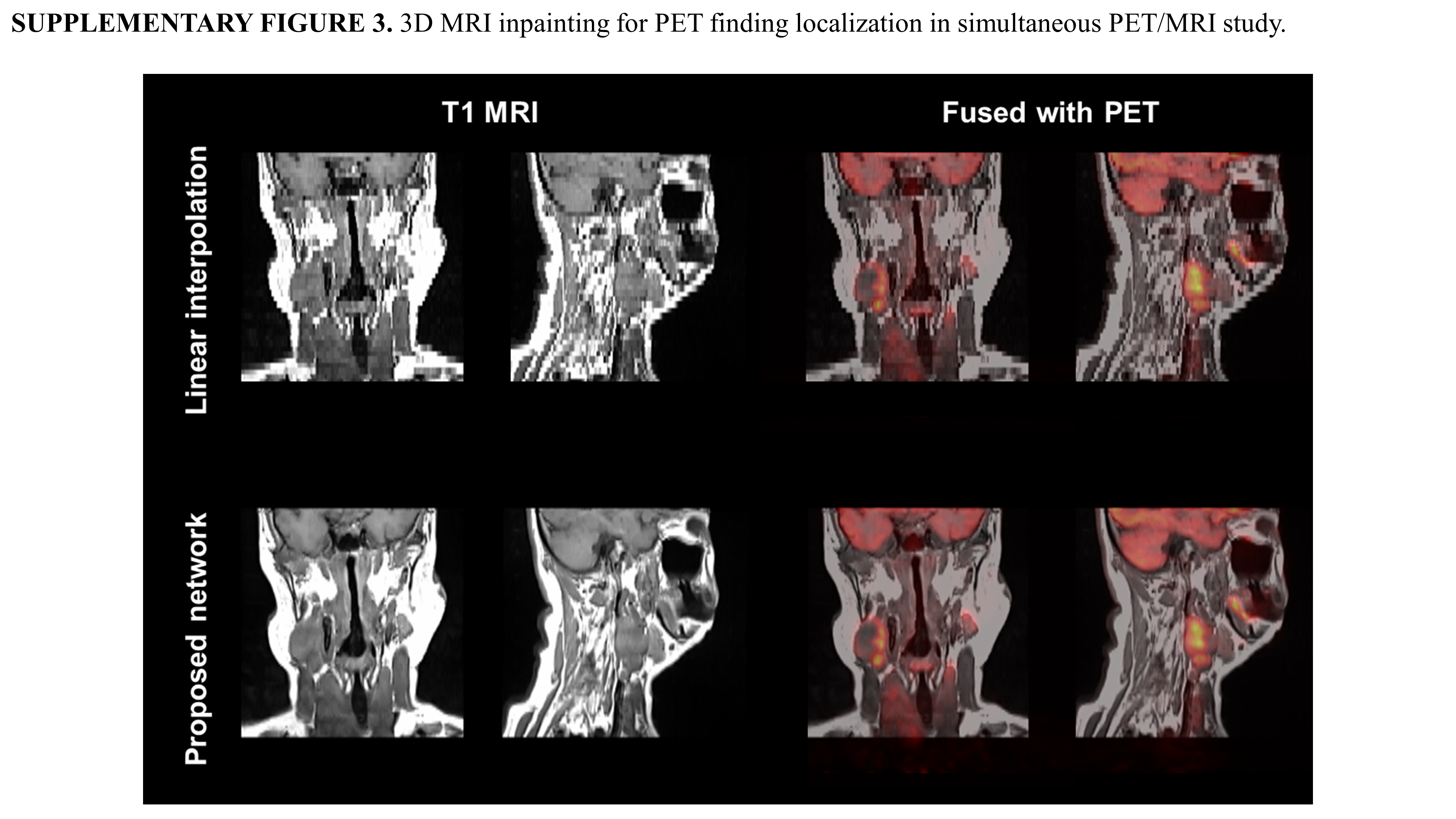

Supplement: Supplementary file 4 — Supplementary Figure 3. [file 41598_2020_80930_MOESM4_ESM.tif]

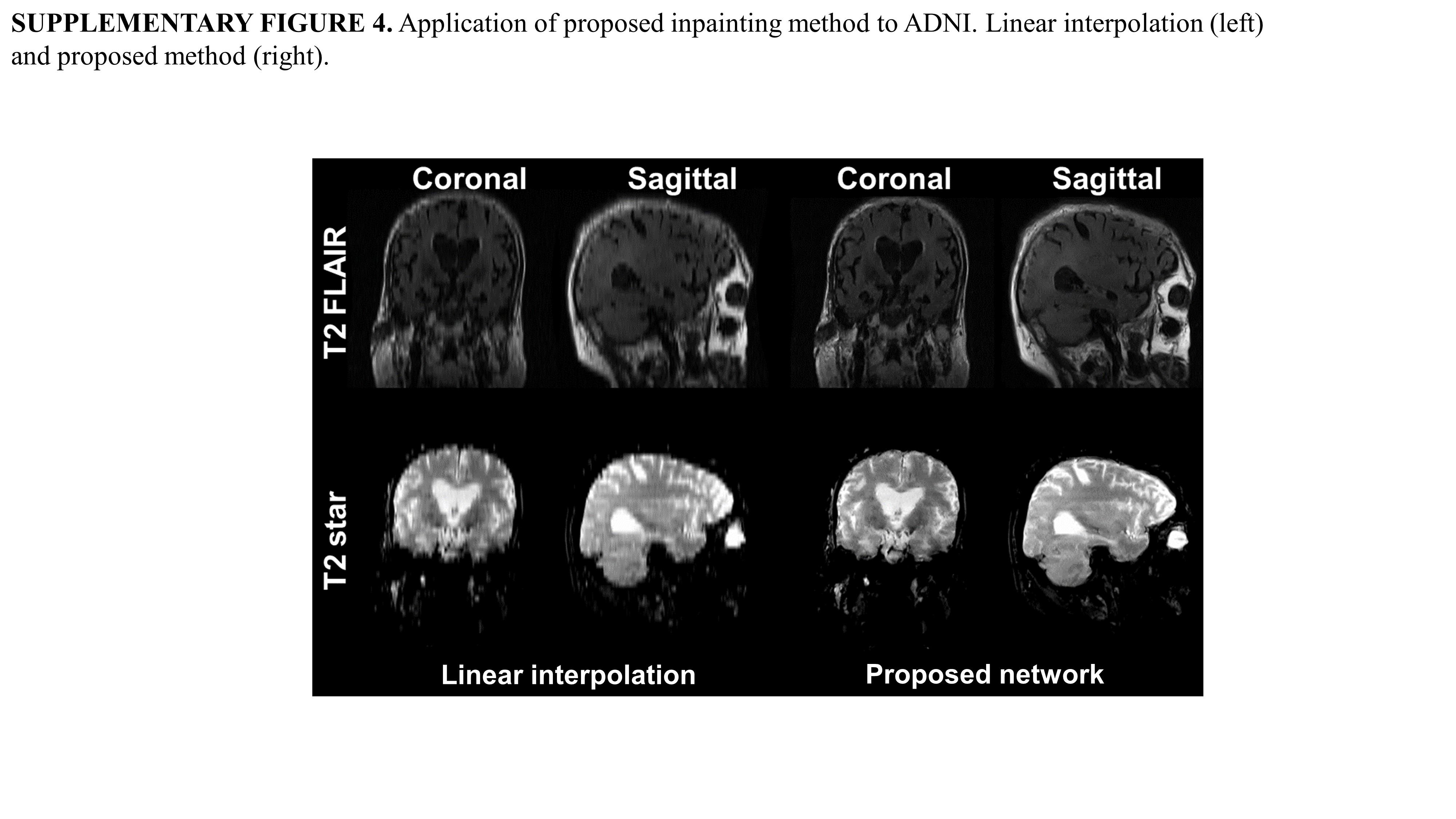

Supplement: Supplementary file 5 — Supplementary Figure 4. [file 41598_2020_80930_MOESM5_ESM.tif]

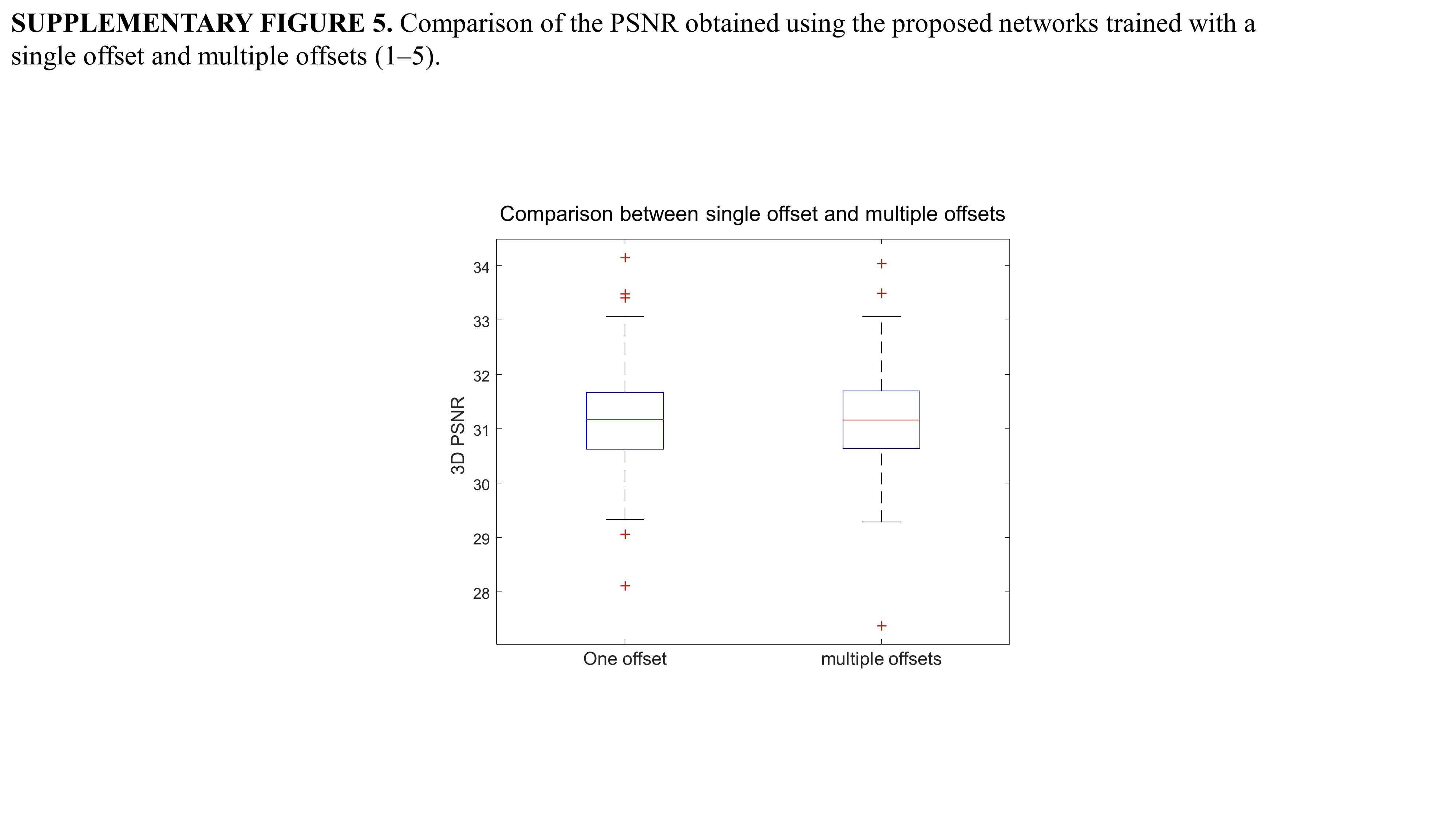

Supplement: Supplementary file 6 — Supplementary Figure 5. [file 41598_2020_80930_MOESM6_ESM.tif]

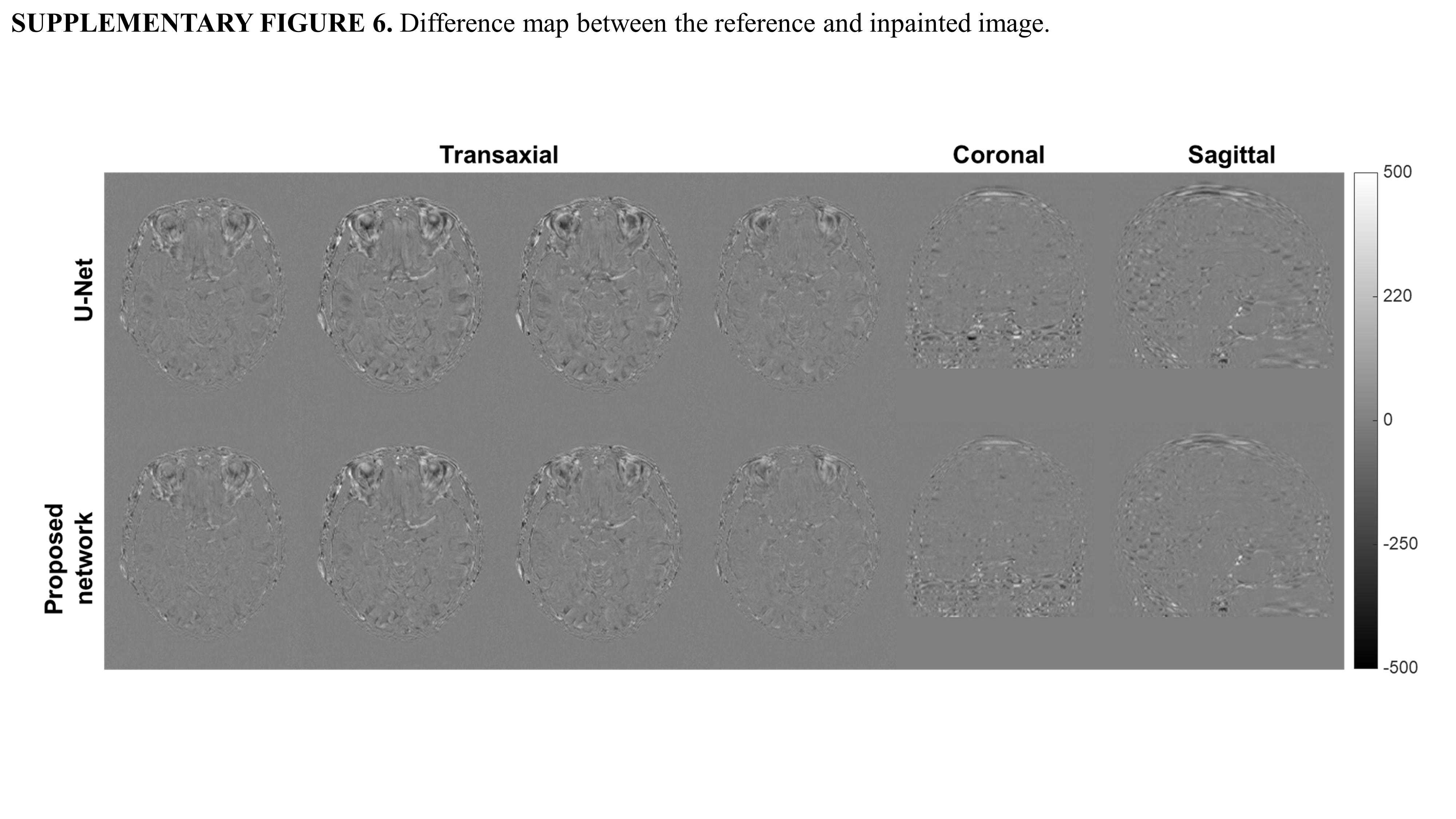

Supplement: Supplementary file 7 — Supplementary Figure 6. [file 41598_2020_80930_MOESM7_ESM.tif]
