# Supplementary material for: Deep learning-Based 3D inpainting of brain MR images
Source: Sci Rep. 2021 Jan 18;11:1673. doi: 10.1038/s41598-020-80930-w (PMC7814079; doi:10.1038/s41598-020-80930-w)
Supplement: Supplementary file 8 — Supplementary Table 1. [file 41598_2020_80930_MOESM8_ESM.pdf]

SUPPLEMENTARY TABLE 1. Network details for generator network.

**Layers**	**Output size**	**Number of channels**	**Convolutional kernels**
Convolution 1	32x32x32	32	3x3x3 conv
		16	1x1x1 conv, 3x3x3 conv
		16	1x1x1 conv, 3x3x3 conv
Dense block 1	32x32x32	*16*	1x1x1 conv, 3x3x3 conv
		16	1x1x1 conv, 3x3x3 conv
		16	1x1x1 conv, 3x3x3 conv
Transition layer 1	16x16x16	*16*	3x3x3 conv
		16	1x1x1 conv, 3x3x3 conv
		16	1x1x1 conv, 3x3x3 conv
Dense block 2	16x16x16	*16*	1x1x1 conv, 3x3x3 conv
		16	1x1x1 conv, 3x3x3 conv
		16	1x1x1 conv, 3x3x3 conv
Transition layer 2	8x8x8	*16*	3x3x3 conv
		16	1x1x1 conv, 3x3x3 conv
		16	1x1x1 conv, 3x3x3 conv
Dense block 3	8x8x8	*16*	1x1x1 conv, 3x3x3 conv
		16	1x1x1 conv, 3x3x3 conv
		16	1x1x1 conv, 3x3x3 conv
Transition layer 1	4x4x4	*16*	3x3x3 conv
		16	1x1x1 conv, 3x3x3 conv
		16	1x1x1 conv, 3x3x3 conv
Dense block 4	4x4x4	*16*	1x1x1 conv, 3x3x3 conv
		16	1x1x1 conv, 3x3x3 conv
		16	1x1x1 conv, 3x3x3 conv
Transition layer 3	2x2x2	*16*	3x3x3 conv
		16	1x1x1 conv, 3x3x3 conv
		16	1x1x1 conv, 3x3x3 conv
Dense block 5	2x2x2	*16*	1x1x1 conv, 3x3x3 conv
		16	1x1x1 conv, 3x3x3 conv
		16	1x1x1 conv, 3x3x3 conv

Transition up layer 1	4x4x4	*16*	3x3x3 deconv
		16	1x1x1 conv, 3x3x3 conv
		16	1x1x1 conv, 3x3x3 conv
Dense block 6	4x4x4	*16*	1x1x1 conv, 3x3x3 conv
		16	1x1x1 conv, 3x3x3 conv
		16	1x1x1 conv, 3x3x3 conv
Transition up layer 2	8x8x8	*16*	3x3x3 deconv
		16	1x1x1 conv, 3x3x3 conv
		16	1x1x1 conv, 3x3x3 conv
Dense block 7	8x8x8	*16*	1x1x1 conv, 3x3x3 conv
		16	1x1x1 conv, 3x3x3 conv
		16	1x1x1 conv, 3x3x3 conv
Transition up layer 3	16x16x16	*16*	3x3x3 deconv
		16	1x1x1 conv, 3x3x3 conv
		16	1x1x1 conv, 3x3x3 conv
Dense block 8	16x16x16	*16*	1x1x1 conv, 3x3x3 conv
		16	1x1x1 conv, 3x3x3 conv
		16	1x1x1 conv, 3x3x3 conv
Transition up layer 4	32x32x32	*16*	3x3x3 deconv
		16	1x1x1 conv, 3x3x3 conv
		16	1x1x1 conv, 3x3x3 conv
Dense block 9	32x32x32	*16*	1x1x1 conv, 3x3x3 conv
		16	1x1x1 conv, 3x3x3 conv
		16	1x1x1 conv, 3x3x3 conv
Convolution 2	32x32x32	1	9x9x9 conv
